# Healthcare Workers and Manual Patient Handling: A Pilot Study for Interdisciplinary Training

**DOI:** 10.3390/ijerph17144971

**Published:** 2020-07-10

**Authors:** Elpidio Maria Garzillo, Maria Grazia Lourdes Monaco, Anna Rita Corvino, Francesco D’Ancicco, Daniela Feola, Dino Della Ventura, Nadia Miraglia, Monica Lamberti

**Affiliations:** 1 Department of Prevention, Abruzzo Local Health Authority, 67100 L’Aquila, Italy; egarzillo@asl1abruzzo.it; 2 Department of Experimental Medicine, University of Campania “Luigi Vanvitelli”, 80138 Naples, Italy; annarita.corvino@unicampania.it (A.R.C.); danciccofrancesco@gmail.com (F.D.); nadia.miraglia@unicampania.it (N.M.); monica.lamberti@unicampania.it (M.L.); 3 Occupational Medicine Unit, University Hospital of Verona, 37134 Verona, Italy; 4 Occupational Physician in Healthcare Setting, 40138 Bologna, Italy; feolad@gmail.com; 5 Department of Neuroscience, Reproductive Sciences and Dentistry, University of Naples Federico II, 80131 Naples, Italy; dinodellaventura@me.com

**Keywords:** healthcare workers, manual patient handling, occupational risk training

## Abstract

Manual patient handling (MPH) is a major occupational risk in healthcare settings. The aim of this study was to propose an MPH training model involving interdisciplinary aspects. A scheduled training program was performed with 60 healthcare workers (HCWs) from a hospital in Naples, Italy, providing training divided into three sections (occupational health—section one; physical therapy—section two; psychosocial section—section three) and lasting six hours. Fifty-two HCWs performed the training session. In section one, a questionnaire about risk perception related to specific working tasks was administered. Section two provided specific exercises for the postural discharge of the anatomical areas most involved in MPH. The last section provided teamwork consolidation through a role-playing exercise. The training program could also be useful for risk assessment itself, as they can examine the perceptions of the specific risk of the various workers and incorrect attitudes and therefore correct any incorrect procedures, reducing exposure to specific risks in the field. This pilot study proposes a training model that explores all aspects related to MPH risk exposure and also underlines the need for standardization of this formative model, which could represent a useful tool for studying the real effectiveness of training in workplaces.

## 1. Introduction

Manual patient handling (MPH) is one of the major occupational risks for healthcare workers (HCWs). According to the 6th European Working Condition Survey (EWCS), several working tasks are performed in lifting or moving people. One explanation could be the recent expansion of the care sector in Europe, where a number of occupations require these types of tasks. According to the European survey, an increase in the percentage of workers involved in MPH (up to 10%) could be observed, and this is the only posture-related risk among those included in the EWCS that is shown to be on the increase. In particular, the percentage of female workers involved in MPH tasks for one-fourth to three-fourths of their working time is 9%, double that of men [1].

These exposure data account for the high percentage of musculoskeletal disorders (MSDs) in some categories of workers. The work-related musculoskeletal disorders (WRMSDs) in nursing workers are well reported in the scientific literature; the mean annual prevalence rates are 55% for low back pain (LBP), 44% for shoulder pain, 42% for neck pain, 26% for upper extremity pain, and 36% for lower extremity pain [2]. The year prevalence of low back pain in nurses has a mean of 70%, and the lifetime prevalence ranges from 35 to 80%. Recurrence rates of low back pain in nurses exceed 70% [3]. This prevalence rate has been found in countries all over the world; moreover, LBP might result in activity limitation for over 50% of HCWs [4,5]. HCWs are committed to several workplace activities (i.e., patient hygiene, the pronation/supination of patients in intensive care units (ICUs), moving patients from beds to stretchers or wheelchairs, etc.) that expose them to a variety of factors and moving geometries associated to LBP development.

This care setting variability and the related range of involved handling tasks are challenges in assessing the safety of MPH conditions and in developing improvement programs, including specific training to all personnel [6].

MPH and the required non-ergonomic postures involve cumulative spinal loads, often associated with lumbar disc structural degeneration and other disorders [7].

**Figure 1:**
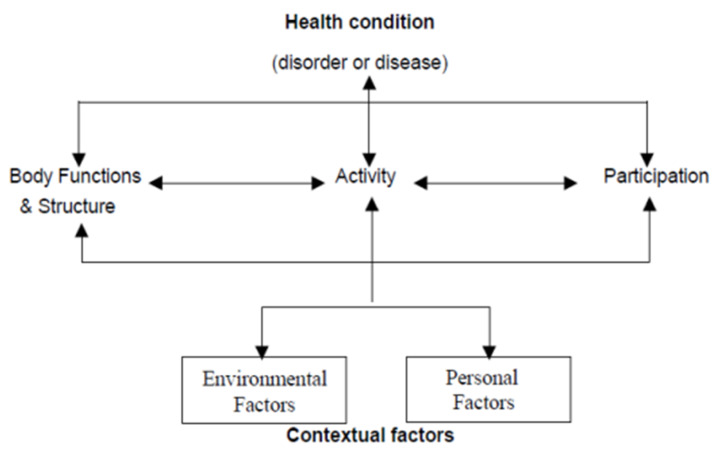
International Classification of Functioning—Paradigm of Overall Health.

According to the WHO biopsychosocial model, health status is granted by the integration of medical and social aspects. As found in the International Classification of Functioning, overall health can be illustrated by the following diagram (Figure 1) [8].

The absence or reduction of physical hazards in the workplace, e.g., when moving patients, is a goal of job quality. All HCWs (i.e., nurses, sanitary aides, helpers and technicians) develop personal characteristics through their experiences, values, attitudes, and biases that have significant effects on their communication with patients. Some HCWs’ beliefs and attitudes are related to a complex relationship between feelings and emotional responses in patient care, work organization, and risk exposure. These aspects need to be deepened in the risk assessment because they could inspire interest in challenging clinical situations and physician self-care, which can improve specific training.

The American Physical Therapy Association (APTA) introduced in 2014 the standards recommendation for the development of safe patient handling (SPH) training programs and issued a position statement on the role of a physical therapist in these programs. Olkowski et al. [9] stated that physical therapists determine the most appropriate handling method for both patients and HCWs and they train HCWs in the use of SPH equipment and practices, participate in SPH programs, and could be involved in SPH policy. Moreover, several studies have investigated the effectiveness of physical training in improving the capabilities for manual handling, often highlighting beneficial effects resulting from customized exercises, in terms of improved physical capacity for manual handling tasks [10,11].

According to Clemes et al., high priority should be given to developing and evaluating multidimensional interventions, incorporating exercise training, conducted in a multidisciplinary way, to promote strength and flexibility, involving a physical therapist during the course and exploring practical exercises that could be performed at home or while working [12].

The culture of safety in the workplace undoubtedly influences the shared perceptions of workers within a specific healthcare setting. Consequently, hospitals must increase their focus on environmental and organizational aspects by incorporating a specific training program, which needs to be administered continuously, and taking care of aspects aimed at creating a safety culture that involves safe patient handling and mobility tasks [13].

Healthcare settings predispose HCWs to psychological effort and stress, making nursing a high-risk occupation. This often results in a deterioration of the relational and teamwork skills that underlie the cooperation and sharing of some risk exposures, such as MPH. Very often, in fact, numerous bedside operations (i.e., therapeutic maneuvers or patient bedside hygiene) require the involvement of multiple operators, simultaneously. Coordination in these operations is necessary for the reduction of risk exposure. An impaired relationship capacity may occur in the risk perception, deteriorating the perception itself. Useful teamwork is therefore the basis for sharing the risk among the operators. Communication and cooperation within an exposed group to MPH, especially for tasks involving the simultaneous activity of two operators, is activated by the relationships that are established between the workers; it is not just simple information sharing, but it is a complex social phenomenon influenced by the emotional, cultural, and social background of the participants and by the context in which it occurs. Moreover, Lee et al. showed that the safety climate in workplaces was the most influential factor associated with safe patient handling behaviors among critical care nurses [14]; a positive organizational safety climate, a people-oriented culture, and ergonomic practices were significant factors for safe patient handling behaviors among hospital nurses [15].

The number of patient-handling activities per day was a hard measure in risk assessment; this risk exposure could be assessed by single questions directly addressed to operators. Various assessment methods have been developed over the years. These methods present several limitations; for example, some aspects of HCWs’ physical work demands, such as non-ergonomic trunk postures that may generate a relatively high mechanical load on the back, were underestimated within some evaluation assessments. HCWs often perform physical tasks that are considered complex, unplanned, and unpredictable.

Many authors recommend that specific MPH training plays a key role in prevention programs related to manual handling [16,17]; these programs might show transfer techniques practice for all staff and provide feedback on the skills of trained staff [18]. In many countries, such as the United States, there is no standardized method for training in MPH, despite the high incidence of injury. In Italy, the MPH training programs are often non-specific and take more care of the legislative and theoretical aspects than the practical ones. In addition, the applied skills are always aimed at training in the use of technical aids, with less impact on the postural gesture linked to the correct movement of the patient. Targeted exercises and physiotherapeutic techniques of postural reprogramming are undoubtedly recommended for LBP treatment, management, and chronicity prevention, more specifically in HCWs exposed to MPH. HCWs who had less education, strength training, and fitness levels had a lower adherence to exercise programs designed based on prevention risk. Motivational strategies should be targeted at these persons, even scheduling specific work training related to MPH exposure risk [19].

However, elaborating this program is complex, as it requires in-depth knowledge about the workplace and the work organization, the shifts carried out, the clinical and psychological conditions of the personnel involved, the risk assessment, and the risk perception that these workers have about their condition. On-the-job training would have a greater impact than, for example, a non-contextualized education. The nature of training, i.e., theoretical, practical, or both, must also be considered to optimize knowledge application and favorize the opportunity of applying knowledge in real settings. Resnick et al. also identified workplace constraints that can hinder the implementation of preventive practices. For instance, participants mentioned that the difficulty of accessing equipment, as well as overcrowded workspaces, particularly for in-home care, complicates the application of the preventive practices they have learned [20]. Other scientific data confirm that the work environment can influence the application of preventive measures [17].

Thus, each specific training program carried out in the workplace should be thoroughly planned and not be exclusively due to legal obligation. Training and education programs are widely adopted as key injury prevention strategies. Training aimed at refuting incorrect attitudes and reconditioning daily operational gestures could be a primary objective of training programs for HCWs exposed to MPH risk, such as the model proposed in this paper.

The aim of this pilot study was to assess the feasibility of an interdisciplinary MPH training model, in order to define a standardized model that can be generally proposed to workplaces.

## 2. Methods

### 2.1. Setting and Study Design

The pilot study was carried out in a Southern Italian hospital (Naples, Italy) in November 2018 and enrolled 60 HCWs. Within the scope of the mandatory training for HCWs exposed to MPH, an innovative interdisciplinary program was created. This training model was proposed and approved by the Hospital Health Management. The program was divided into three sections with a total duration of 6 h, according to specific Italian regulations. Each module lasted 2 h and consisted of theoretical and practical parts, focusing on three aspects: (a) acquiring theoretical knowledge and updates on patient-centered handling according to the WHO BioPsychosocial Health Model; (b) learning manual, technical, and practical skills for the dynamic patient–environment–worker interaction and physical effort evaluation, through techniques of global postural reprogramming according to the Mézières method; (c) improving the relational and communicative skills within the working group.

**Figure 2:**
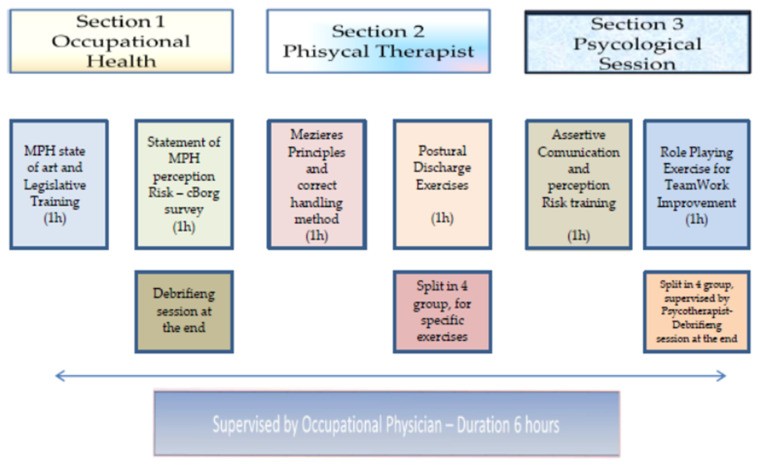
Training program scheme.

A scheme of the training program is summarized in Figure 2.

An occupational physician coordinated section one. This section was developed in two parts. The first part, lasting 60 min, was dedicated to a frontal lesson in which, after a brief mention of the biomechanical overload problems in the workplace and the main related risks, the assessment of the current MPH and the inanimate loads risk in the hospital were illustrated. In the next 60 min, the HCWs filled out a form on the risk perception about MPH. It was a self-administered questionnaire that consisted of a free grid, in which the workers had to represent three of the worst handling conditions they had ever perceived; each free item was then accompanied by a score according to the Borg c10 scale, appropriately illustrated [21]. For each department, the state of the art concerning biomechanical risk assessment was described. The authors used this training moment to illustrate to all participants the results of the biomechanical risk assessment, carried out through validated methods, such as the “Lift Index” by National Institute for Occupational Safety and Health (NIOSH) for load handling and the “Movement and Assistance of Hospital Patients” (MAPO) index for MPH risk assessment; subsequently, after collection of the Borg scale results, a comparison was made between the assessed risk using NIOSH/MAPO and the perceived risk evaluated on-site using the Borg scale. This comparison was then commented on via an interactive approach between the teacher and learners. After the end of the training program, all these data were discussed with the hospital’s Health and Safety Department and included in the risk assessment document as a participatory approach to risk assessment in this issue.

A physiotherapist coordinated section two of the training program. At first, the main techniques of patient handling were illustrated; afterwards, the HCWs were trained on the aforementioned correct handling maneuvers [22] as well as the postural/overload unloading training through some of Mézières’ basic global postural reeducation techniques.

The last module (section three) was conducted by a psychotherapist, who focused on the theoretical part of the problems inherent in risk perception and on the practical part related to assertive communication and role playing for teamwork improvement [23].

Data were reported according to the STROBE statement guidelines for reporting observational studies [24].

### 2.2. Training Model Evaluation

To evaluate the proposed model, several aspects were simultaneously taken into account. First, a final satisfaction questionnaire, developed by the authors, was administered to all participants at the end of the course. This survey consisted of six questions, each with five closed answers (with five items—strongly agree, agree, neutral, disagree, strongly disagree) and three open-ended questions. Another variable was the adherence to the interdisciplinary training program vs. standard (HCWs participation percentage).

Last but not least, the management elaborated a cost-effectiveness analysis of the training program proposed.

### 2.3. Ethics

This pilot study was conducted within the mandatory training workers’ program according to Italian law [25]. The research was performed following the ethical standards laid down in the 1964 Declaration of Helsinki and its later amendments.

### 2.4. Statistical Analysis

Data were analysed using SPSS ver. 21 (IBM Corp. Released 2012. IBM SPSS Statistics for Windows, Version 21.0. Armonk, NY, USA: IBM Corp.). Descriptive analysis and continuous variables were given as the mean ± standard deviation (SD), and categorical variables were given as the absolute value and relative frequency.

## 3. Results

The total sample enrolled for training was made up of 60 HCWs. Eight workers did not complete the course (three were sick at the scheduled date, and five of them were not available for personal reasons and preferred to be rescheduled for the next training course, performed in the regular way). Twenty-five men and 27 women (mean age 49.4 (SD ± 7.2) and 45.9 (SD ± 8.8), respectively) were finally engaged. The cohort group had a mean job seniority of about 24.6 years (SD ± 8,1) and a median age of 26.5 years (range: 7–38). Enrolled workers came from several hospital departments involved in MPH, such as surgery (orthopedics, bariatric surgery, etc.), maternal-fetal medicine, medicine and health services, and physiotherapy, where bedridden patients are hospitalized.

### 3.1. Training Program—Occupational Medicine Section

**Figure 3:**
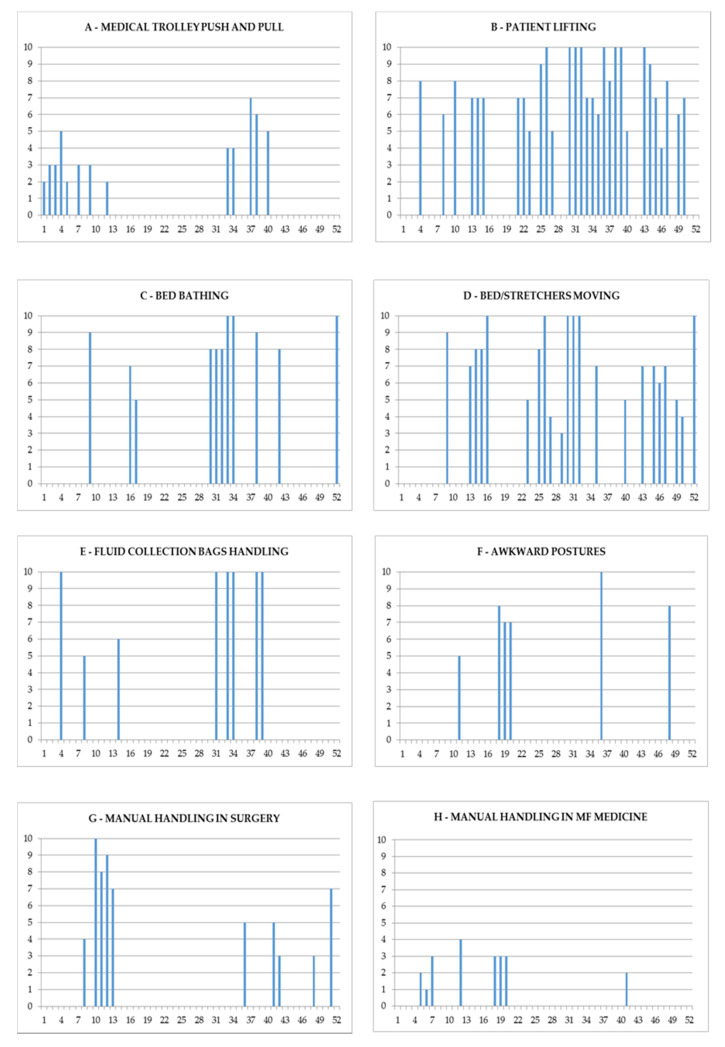
Answers provided by healthcare workers (HCWs) to the questionnaire. A–H each box refers to a task identified by the HCWs as overloading. The abscissa axis shows the reference number of the HCW. The ordinate axis shows the value of the Borg scale (Borg CR-10) attributed by each worker to the task.

Figure 3 shows the questionnaire results. Letters from A to F show the Borg’s scale scores attributed to the task categories by the subjects enrolled in the study. The most represented tasks include patient lifting (*n*.30 answers; Borg CR-10: mean: 7.66 (±1.80); median: 7; range: 4–10), bed/stretcher transferring (*n*.22 answers; Borg CR-10: mean: 7.27 (±2.25); median: 7; range: 3–10), and patient bed hygiene (*n*.11 answers; Borg CR-10: mean: 8.36 (±1.50); median: 8; range: 5–10), which are tasks perceived as heavier.

Overall, activities such as the handling of medical/emergency trolleys and materials supply, which are involved in inanimate load moving, are considered to pose less risk within the sample examined (n.13 answers; Borg CR-10: mean 3.76 (±1.58); median: 3; range: 2–7). Lastly, for the staff of the maternal-foetal ward (Figure 3-H) there is a lower perception of the MPH risk, given the small weight of the patients, compared to the movement of inanimate loads (as incubators).

### 3.2. Training Program—Physiotherapy Section

**Figure 4:**
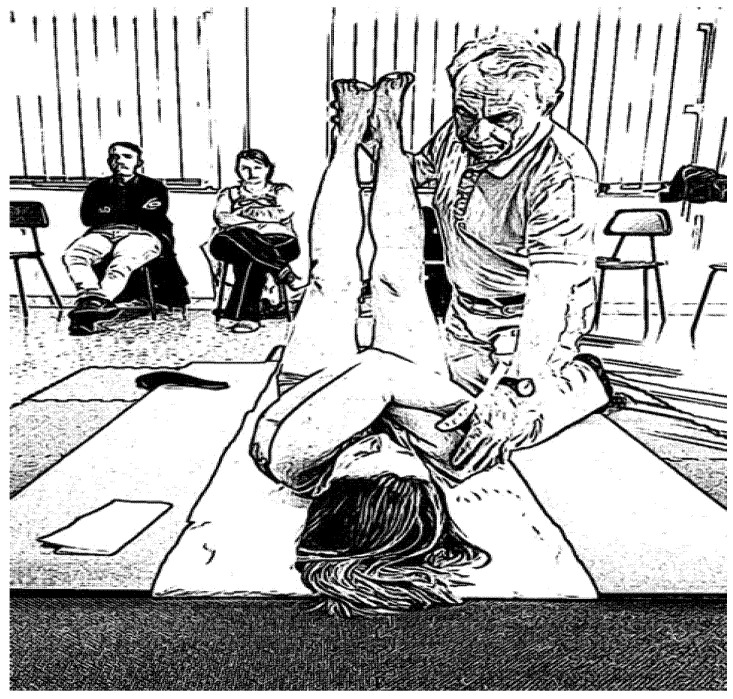
An example of an exercise performed by the physiotherapist during section two of the training. Lumbar stretching according to the Méziéres method.

**Figure 5:**
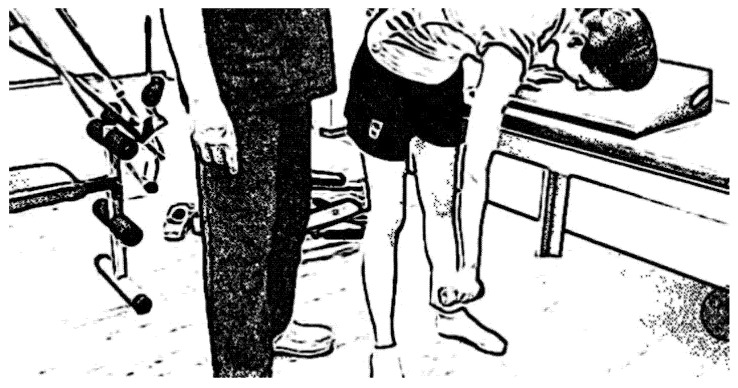
An example of an exercise performed by the physiotherapist during section two. Codman’s (pendulum) exercises.

This program was carried out by a physiotherapist and lasted a total of two hours. Participants were divided into four groups of 13 people. The first part (1 h) was characterized by frontal training on the general principles regarding the correct bedside posture gesture and the correct illustrated movements of the patients following specific manuals [22]. Afterwards, for the practical part, the postural discharge exercises were illustrated and conducted according to the postural reprogramming theories in the Mézières method. These exercises can be resumed cyclically within specific routine training that will be carried out autonomously in the hospital or at home. Figure 4 and Figure 5 show two examples of taught activities for postural discharge, respectively, for the lumbar spine, which is most affected by biomechanical overload [26], and for the shoulder-humeral girdle.

### 3.3. Training Program—Psychological Section

The last section focused on the need to correctly perceive the risk and to establish useful teamwork in order to reduce any incorrect practices in terms of patient handling. First, a theoretical session was carried out (1 h); the importance of risk perception and the differences between the perceived risk and the real working environment were illustrated. The theory of assertiveness [27] was also introduced in order to explain the next session. The practical session involved the participants divided randomly into four teams, who had been assigned a single specific objective related to the role-playing activity. According to the proposed scenario, each worker had to imagine themselves as part of the crew of a spacecraft having landing problems, one of the solutions for which was to remove part of the load of the spacecraft, consisting of necessary goods such as water, oxygen, freeze-dried food, clothing, and drugs. Each group was asked to reach a unanimously shared conclusion about the elimination of the objects described above, each supposedly indispensable. Finally, the three groups had to come to a unanimous agreement, showing a good level of teamwork. One group showed critical issues by not reaching a shared decision. A debriefing session to finally discuss the results of each group was carried out.

### 3.4. End of Course Evaluation

**Figure 6:**
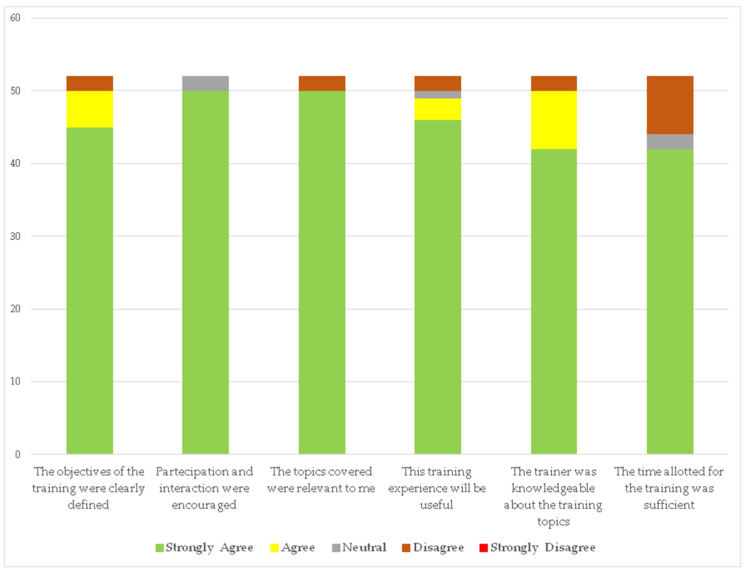
Satisfaction questionnaire results.

As shown in Figure 6, the feedback was overwhelmingly positive. Several suggestions coming from the open-ended questions concerned the course duration, in particular regarding section two, as emerged from open questions (six HCWs directly requested longer training by the physiotherapist).

As for the training participation, only three of the 55 workers available (among 60 HCWs involved, five were not available for personal reasons) expressly preferred to enrol in the standard training program, with an adherence rate of 94.5%.

Finally, the hospital management carried out a cost-effectiveness analysis. Although the final direct costs were higher than the standard course previously conducted, due to the need for a multidisciplinary teaching team and the division of workers into small groups, the management reported that the innovative methodological approach was sustainable as well as the standard one.

## 4. Discussion

Healthcare organizations worldwide are increasingly focusing on strategies to create a safe patient handling culture in workplaces. Nonetheless, the WRMSD rate among HCWs continues to increase despite efforts concentrating on valid MPH training and education. European regulations legislate the necessity of specific training programs to inform all workers about the risks to which they are exposed, the working procedures and standards that must be observed, and personal protective equipment and their specific use. Thus, specific training is, in fact, an important aspect of occupational health and safety and represents a critical factor in prevention processes within organized structures, including healthcare settings. Ziam et al. showed some critical issues: the basic MPH education taken at college and university, as well as on-the-job training, appears inadequate due to lack in context and duration of training performed; WRMSD prevention training is not a ‘concern’ and is rather rare in the real job context [28].

It is therefore clear that MPH training could play an important role in workplace prevention. Training programs could also be useful for risk assessment itself, as they could examine the perceptions of the specific risk of the various workers and incorrect attitudes and therefore correct any uncorrected procedures, reducing the exposure to specific risks in the field. However, the training programs are not standardized worldwide, both in terms of content and training approach.

This pilot study allowed us to experiment with a multidisciplinary approach focused on several preventive steps, such as the assessment of risk, its perception, and some “physical therapy strategies” for reducing WRMSDs.

### 4.1. Training Program—Occupational Medicine Section

In our opinion, the results from this training section should be useful to produce a better contribution to risk assessment; on the basis of the data provided by the questionnaire, it was concluded that the most perceived risk was the manual handling of the non-collaborating patient, according to assessment results; the operators involved in this type of movement (general medicine, post-surgical department, and ICUs) agreed to assign a score of 10 to the Borg c10 scale. Moreover, the transposition of the bedridden patient (for example, from the bed to the stretcher) and the patients’ bed hygiene are perceived as heavy. The MPH tasks have a variable movement geometry, often attributable to one of three groups, i.e., lifting, repositioning, or turning, with different levels of peak net torque and compression at the lumbar, particularly at the L4/L5 joint. Patient lifting produces spinal compressive force and anterior/posterior and lateral shear forces at the L4/L5 and L5/S1 disks, as shown by biomechanical evaluations [29].

By highlighting the risk perception related to a single MPH task, it might be possible to lead a risk assessment review and to modify some incorrect attitudes held by employees in a real job context. User comfort and perceived physical exertion are necessary for a full risk assessment. Visual analogue scale (VAS) and Borg’s physical effort rating scale are useful for a quick assessment of the effects of handling and working posture [30]. These scales must be included as an integral part of MPH risk assessment for their feedback function in work settings. The rating of perceived exertion in the low back that includes the kinematics of the lumbopelvic–hip complex during patient transfer needs to be measured using Borg’s CR-10 scale [31]. Our results are consistent with literature data [32]. It should be noted that, with regard to the percentage of workers who had not indicated patient lifting as critical, most of them came from departments in which these tasks were assessed as low risk (e.g., maternal-fetal and health services departments).

In this occupational context of the “uncertainty” of evaluation methods, the role of training seems to be a main aspect of prevention interventions. Smedley et al. reported that improving the manual-handling training program and extending the use of patient-handling equipment in the wards “may not have the desired effects on work methods or on the rates of back symptoms, at least in the short term” [33]. However, in our opinion, these training programs need to be customized in order to be effective, according to the operational needs of the various hospital departments, the handling aids made available to the staff, the characteristics of the patients to be treated, and, last but not least, the awareness of HCWs to risks related to the handling tasks. MPH must have an interdisciplinary approach, as it must be safe for both the operators and the patients themselves. In a new interpretation of patient handling, the transition from operator-centered to person-centered handling eliminates factors that hinder the autonomy of the patient in the therapeutic, organizational, and relational environment, and the use of autonomy aids effectively eliminates or reduces the demand for MPH.

### 4.2. Training Program—Physiotherapy Section

Training programs are often focused on education about body mechanics and patient handling techniques and remain the preferred prevention approach to reduce LBP in HCWs [34]. In some literature reviews [35,36], specific training in handling techniques is included as worker-directed preventive measures, such as training in the proper use of patient handling equipment and the presence of peer leaders.

As shown in the final satisfaction survey, the practical session conducted by the physiotherapist represents one of the most valuable training moments in the overall training program.

The model of training programs and their content are often centered around teaching patient handling techniques only. The approaches may vary: training may also be theoretical regarding legislative orientation, biomechanical and ergonomic principles, or equipment use, with just a short practical session concerning moving techniques. The literature shows that this method, when used in isolation, not focusing on practical sessions, has consistently failed to reduce MSDs in nursing staff [37]. Other training programs directly and actively involve all participants focusing on physical exercises such as muscle training, stretching, and endurance. These exercises can be done at home or in the workplace [38]. To date, these training programs can vary significantly from one healthcare setting to another in terms of frequency and duration.

### 4.3. Training Program—Psychological Section

The scientific literature suggests that individual workers’ safety practices are affected by organizational and psychosocial job factors. One of the most prominent issues regarding MPH in healthcare workplaces is the lack of support from colleagues, and this represents a significant barrier to the implementation of MSD prevention practices. Teamwork often depends on the working conditions in each setting.

The emergency situations could represent a stressful job setting that often does not change behavioral attitudes, which often leads to unsuitable postural gestures. In this context, incorrect risk perceptions are also associated, leading to the persistence of incorrect patient moving. Garg et al. [34] have reported that nurses experience high stress on the shoulder and lower back during manual patient lifting and transfer. Performance and motivations are aspects that should be included in specific training related to risk exposure. In particular, the workers’ shared perceptions about their safety in the workplace have been associated with higher incidences of safe work practices in various healthcare settings [39,40].

The qualitative aspects of the communication exchange often escape awareness, compromising the effectiveness of the interaction. Assertive communication is a full and complete manifestation of oneself, functional with respect to the legitimate affirmation/expression of one’s rights, interests, feelings, and beliefs, avoiding the violation or denial those of others, without anxiety or guilt. Two meanings coexist here: (1) to affirm and to make explicit one’s opinions and attitudes and (2) to remain committed to positively resolving problematic situations.

Training on a specific risk factor, such as MPH, should also examine the aspects of relational competence within a complex relational setting in the workplace. The illustration of the assertiveness theory was the basis for proposing the practical session of role-playing, according to the methods described in the results. Indeed, role-playing allows for observation of the ability to problem-solve and demonstrate good cooperation during interactions.

Roleplay often indicates how difficult it can be to assess a shared decision regarding moving a patient in a routine scenario, or in an emergency. In addition, role-playing in supervised groups is a helpful tool to promote reflection and insight not only for the HCWs’ roles, but also for peers observing critical issues at the end of group sessions [41]. In our experiment, a debriefing session conducted with the class after the role-playing scenario provided positive feedback. This clinical role play facilitates teamwork, which increases involvement, self-efficacy, and empathic abilities in healthcare settings and in shared risk exposures.

There is a lack of empirical evidence regarding role-playing and its effectiveness in teamwork improvement. However, the agreement within the groups was considered as an index of the achievement of the planned goal.

However, there are critical issues regarding specific MPH training. A 2010 systematic review found that manual handling training is largely ineffective in reducing LBP, with considerable evidence supporting the fact that the principles learnt during training are not applied in the work environment [42]. In our opinion, this statement could be related to the type of work training. In this work, we showed that there is no standard program for MPH training. This pilot study proposes a model that can be standardized and highlights how the three training moments, i.e., occupational medicine, the physiotherapist, and the session dedicated to improving risk perception and teamwork activities, must be considered as a unique formative moment that improves overall MPH, as it explores all the dynamics (legislative, physical, and psychological) connected to this risk exposure.

Moreover, some studies have demonstrated that training is more cost-effective than engineering controls but that the overall effectiveness of training remains low [43]. In our opinion, this is attributable to a lack of standardization among training courses, which are still not sufficiently setting-oriented. This drawback, furthermore, does not allow in-depth studies on the real outcomes of specific training, because MPH training is overly heterogeneous, even within the same nation.

The scheduling of specific HCWs training should be customized for groups of workers and not generically provided by occupational physicians or health departments inside hospitals. They should also directly involve the training of all staff. It is important to devote adequate time to education, prioritizing HCWs’ occupational health and ensuring that the workforce is adequately trained and healthy [44].

### 4.4. Strenght and Weaknesses

This pilot study supports the feasibility of an interdisciplinary didactic approach, balanced in three phases that explore all aspects of risk exposure and that can be easily reproduced in a healthcare setting. Our training program proposal shows that the standardization of MPH model training could be useful to better study several outcomes, such as cost-effectiveness, the WRMSD prevention rate, and workers’ overall on-the-job comfort. Although the final direct costs were higher but sustainable, as already reported, it should be considered that, in this experience, all the teachers were external consultants to the hospital, due to a lack of appropriate staff within the hospital. However, in medium and large healthcare companies, internal professionals could be recruited, thus significantly reducing the costs incurred.

In addition, the participants’ satisfaction and the possibility of modifying incorrect individual work attitudes induced the health management department to approve the continuation of this training program in the same way for subsequent courses. Having the same training model for a long observation period will allow the enterprise to directly monitor all the outcomes of interest, including, above all, the WRMSD prevention rate.

The main limit of the study lies in the objectification of the end results, in particular regarding sections two and three of the training programs. It is necessary to use this proposed approach to conduct prospective longitudinal studies in order to reinforce these final results.

## 5. Conclusions

Interdisciplinary MPH training was found to play a key role in the application of MSD prevention and needs to be adapted according to the individual, the organization, and the context. Indeed, it is fundamental that WRMSD prevention measures are contextualized to each workplace, offering training adapted to different risk perceptions, clinical conditions, and teamwork levels. This pilot study offers an innovative training model underlining the need to standardize this specific aspect of occupational health so as to better study the outcomes in terms of effectiveness and feasibility. In our opinion, for this achievement, the proposed training program might be shared by international scientific community.

## Abbreviations

HCW	health care workers
LBP	low back pain
MPH	manual patient handling
WRMSDs	work-related musculoskeletal diseases
SPH	safe patient handling
WHO	World Health Organization
